# FoxO1 interacts with transcription factor EB and differentially regulates mitochondrial uncoupling proteins via autophagy in adipocytes

**DOI:** 10.1038/cddiscovery.2016.66

**Published:** 2016-10-03

**Authors:** Longhua Liu, Zhipeng Tao, Louise D Zheng, Joseph P Brooke, Cayleen M Smith, Dongmin Liu, Yun Chau Long, Zhiyong Cheng

**Affiliations:** 1 Department of Human Nutrition, Foods and Exercise, Virginia Tech, Fralin Life Science Institute, College of Agriculture and Life Science, Blacksburg, VA, USA; 2 Department of Biochemistry, Yong Loo Lin School of Medicine, National University of Singapore, Singapore

## Abstract

Mitochondrial uncoupling proteins (UCPs) are inducible and play an important role in metabolic and redox homeostasis. Recent studies have suggested that FoxO1 controls mitochondrial biogenesis and morphology, but it remains largely unknown how FoxO1 may regulate mitochondrial UCPs. Here we show that FoxO1 interacted with transcription factor EB (Tfeb), a key regulator of autophagosome and lysosome, and mediated the expression of UCP1, UCP2 and UCP3 differentially via autophagy in adipocytes. UCP1 was down-regulated but UCP2 and UCP3 were upregulated during adipocyte differentiation, which was associated with increased Tfeb and autophagy activity. However, inhibition of FoxO1 suppressed Tfeb and autophagy, attenuating UCP2 and UCP3 but increasing UCP1 expression. Pharmacological blockade of autophagy recapitulated the effects of FoxO1 inhibition on UCPs. Chromatin immunoprecipitation assay demonstrated that FoxO1 interacted with Tfeb by directly binding to its promoter, and silencing FoxO1 led to drastic decrease in Tfeb transcript and protein levels. These data provide the first line of evidence that FoxO1 interacts with Tfeb to regulate autophagy and UCP expression in adipocytes. Dysregulation of FoxO1→autophagy→UCP pathway may account for metabolic changes in obesity.

## Introduction

Obesity is one of the most pressing health concerns in the US.^1–3^ The growing epidemic of obesity has been attributed largely to modern lifestyle characteristic of energy overconsumption and physical inactivity.^3,4^ As such, the strategies limiting energy intake or increasing energy expenditure have been proposed for obesity prevention.^3–5^ Mitochondria play a central role in cellular energy homeostasis.^3,6–8^ In particular, induction of mitochondrial uncoupling protein (UCP) in mice promotes energy dissipation and protects against obesity, while genetic UCP deficiency causes obesity.^5,9,10^ In line with these findings, UCP polymorphisms have been increasingly reported in obese humans,^11,12^ and adipose UCP gene expression is significantly lower in morbidly obese people than in lean individuals.^13^ These studies suggest that dysregulation of UCPs contributes to development of obesity, and understanding the mechanism of regulation of UCPs in adipocytes may lead to new options for obesity prevention and treatment.

UCPs are a family of mitochondrial transporters (or mitochondrial anion carriers) located in the inner membrane.^14,15^ In adipocytes or adipose tissue, three isoforms of UCP have been identified, UCP1, UCP2 and UCP3, although their expression levels vary.^14–18^ UCP1 is primarily expressed in brown adipose tissue, and it uncouples mitochondrial respiration from ATP production/oxidative phosphorylation, dissipating energy in the form of heat.^14,15^ Under certain conditions (e.g., cold exposure), UCP1 expression in white adipocytes can be significantly induced, leading to a browning phenotype.^17^ UCP2 and UCP3 share amino acid identity with UCP1 (59 and 57%, respectively), and their function in mitochondrial uncoupling is still under investigation.^14,15,18^ Although some studies suggested that UCP2 and UCP3 were proton channels like UCP1, others regarded them as ion channels that limit the production of reactive oxygen species, and export toxic fatty acid anions and peroxides from mitochondrial matrix.^14,15,18,19^

FoxO1 is a transcription factor that regulates mitochondrial function and adipocyte differentiation.^2,20–23^ Activation of FoxO1 in liver alters mitochondrial biogenesis, morphology and function in the insulin resistant mice, while genetic ablation of FoxO1 significantly normalizes mitochondria and metabolism.^21,24^ In adipocytes, silencing FoxO1 with specific antagonist or siRNA potently inhibits cell differentiation and lipid accumulation, accompanied with changes in expression of mitochondrial respiration chain proteins.^2,22,23^ Recently we found that FoxO1 controlled lipid droplet growth and adipose autophagy, the cellular process that has been implicated in adipocyte differentiation.^25–29^ Moreover, genetic and pharmacological inhibition of autophagy leads to browning of white adipose tissue, characteristic of increased UCP1 expression.^26–29^ However, it is unknown how mechanistically FoxO1 regulates autophagy and other UCPs (i.e., UCP2 and UCP3). In the present work, we show that FoxO1-mediated autophagy upregulates UCP2 and UCP3 in adipocytes but downregulates UCP1. Mechanistically, FoxO1 interacted with transcription Factor EB (Tfeb), a key regulator of autophagosome and lysosome,^30^ by directly binding to the promoter and regulating its expression.

## Results

### Expression patterns of UCPs during adipocyte differentiation

**Figure 1:**
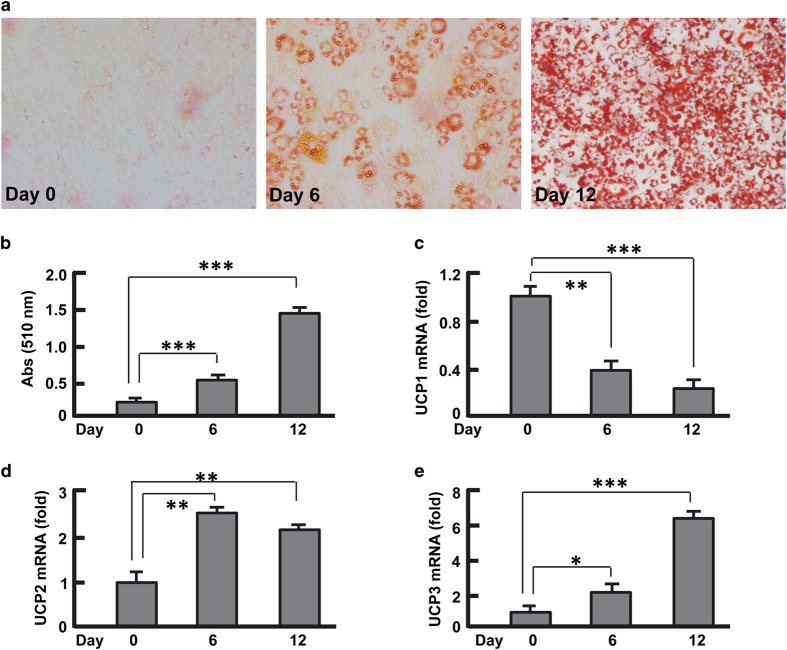
Expression of UCPs during 3T3-L1 adipocyte differentiation. (**a** and **b**) Measurement of lipid accumulation during adipocyte differentiation. The cells were cultured and differentiated as described in Materials and methods section, and lipid accumulation was measured by oil red O staining (**a**) and absorbance at 510 nm (**b**). (**c **and **d**) qPCR analysis of UCP1 (**c**), UCP2 (**d**) and UCP3 (**e**) during adipocyte differentiation. Results were presented as mean±s.d.; *n*=3–4; *P<0.05; **P<0.01; ***P<0.001.

Following an established protocol, we cultured 3T3-L1 preadipocytes and induced cell differentiation.^2,31^ Maturation of adipocytes was paralleled with significant lipid accumulation as measured by oil red O staining and spectrophotometric reading at 510 nm (Figures 1a and b).^2,32,33^ Interestingly, the expression of UCP1, UCP2 and UCP3 showed distinctive kinetics during adipocyte differentiation (Figures 1c–e). In contrast to UCP1 that underwent downregulation (Figure 1c), UCP2 and UCP3 were upregulated drastically (Figures 1d and e). These data support the notion that upregulation of UCP1 counteracts lipid accumulation in adipocytes,^34,35^ and that UCP2 and UCP3 are required for lipid metabolism.^14,15,19,36^

### Inhibition of FoxO1 reversed the coordinated expression of UCPs in adipocytes

**Figure 2:**
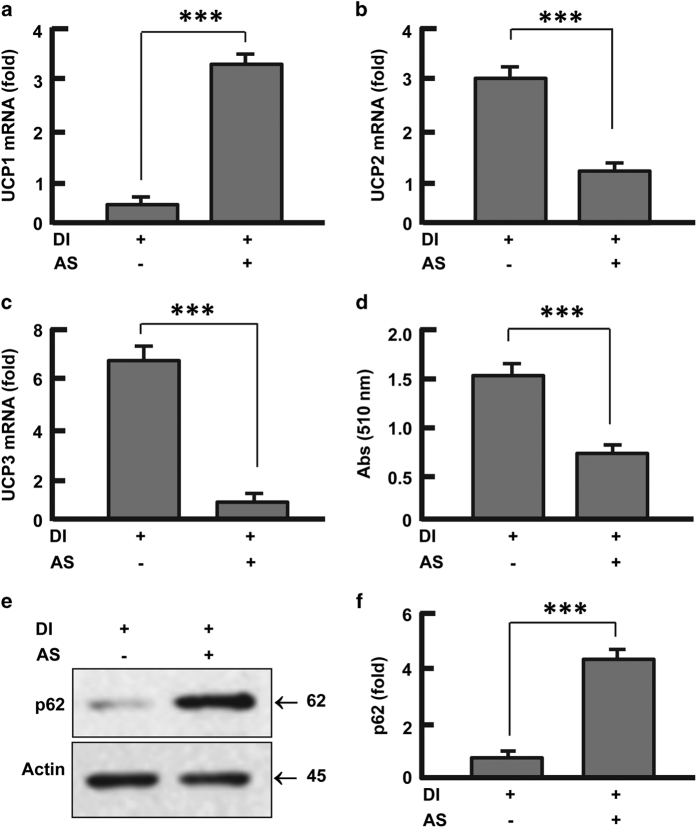
Effects of FoxO1 inhibition on UCPs and autophagy. (**a**) Inhibition of FoxO1 upregulated UCP1. (**b**) Inhibition of FoxO1 down-regulated UCP2. (**c**) Inhibition of FoxO1 down-regulated UCP3. (**d**) Inhibition of FoxO1 prevented lipid accumulation in adipocytes. (**e **and **f**) Inhibition of FoxO1 attenuated autophagy (p62 degradation). The cells were cultured and treated (days 0–12) as described in Materials and Methods section. DI, differentiation induction; AS, AS1842856 (0.1 μM). Results were presented as mean±s.d.; *n*=3–4; ***P<0.001.

FoxO1 regulates mitochondrial morphology and biogenesis,^21,24^ but it remains largely unknown how FoxO1 is related to mitochondrial UCPs. Upon inhibiting FoxO1 during differentiation with a specific antagonist AS1842856,^37^ we found that the coordinated expression of UCP1, UCP2 and UCP3 was significantly disrupted in 3T3-L1 cells (Figure 2, in comparison with). A threefold increase in UCP1 expression was induced by the treatment with AS1842856 (*P*<0.001; Figure 2a). In contrast, inhibition of FoxO1 markedly reduced the expression of UCP2 (by 58%, *P*<0.0001; Figure 2b) and UPC3 (by 87%, *P*<0.0001; Figure 2c). These changes were associated with a drastic suppression of adipocyte differentiation, leading to ~50% reduction of lipid accumulation in the adipocytes (*P*<0.001; Figure 2d). In addition, AS1842856 resulted in a marked inhibition of autophagy (Figures 2e and f; Supplementary Figure 1). Given that UCP1 can be induced by modulation of autophagy,^26–29^ the inhibition of autophagy by AS1842856 may account for the altered UCPs in the adipocytes.

### Suppression of autophagy recapitulated the effects of FoxO1 inhibition on UCPs

**Figure 3:**
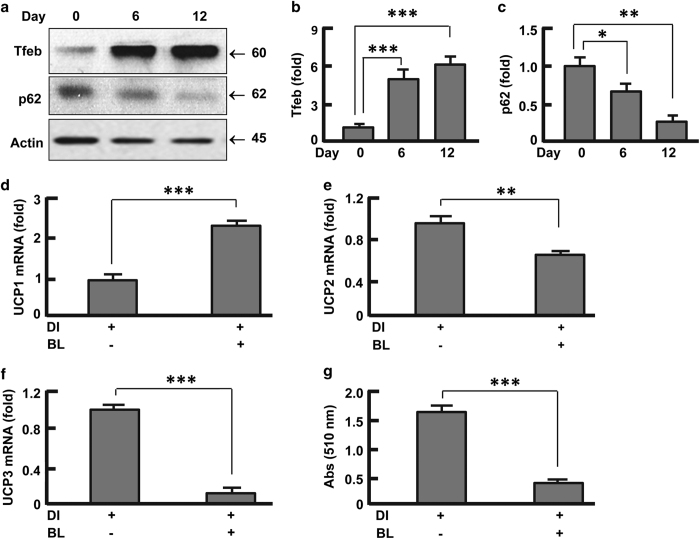
Autophagy was required for coordinated expression of UCPs in adipocytes. (**a**–**c**) Western blot (**a**) and densitometric (**b **and** c**) analysis of Tfeb and p62 suggested that autophagy was upregulated during adipocyte differentiation. (**d**) Effects of autophagy inhibitors bafilomycin A1 and leupeptin on UCP1 expression. (**e**) Effects of bafilomycin A1 and leupeptin on UCP2 expression. (**f**) Effects of bafilomycin A1 and leupeptin on UCP3 expression. (**g**) Effects of bafilomycin A1 and leupeptin on lipid accumulation. The cells were cultured and treated as described in Materials and Methods section, and the treatment with autophagy inhibitors was conducted on days 0–12. DI, differentiation induction; BL, bafilomycin A1 (4 nM) and leupeptin (0.4 μg/ml). Results were presented as mean±s.d.; *n*=3–4; *P<0.05; **P<0.01; ***P<0.001.

To examine the role of autophagy in UCP regulation, we measured kinetics of autophagy during adipogenesis (Figure 3, Supplementary Figure 1). Adipocyte differentiation was accompanied with a gradual reduction of p62 (Figures 3a–c), the protein that was exclusively degraded by autophagy.^25,38,39^ This change was concurrent with upregulation of Tfeb (Figures 3a and b), the transcription factor that regulates both autophagosome and lysosome,^30^ supporting the notion that autophagy is induced during adipogenesis.^25,28,40^ To test whether autophagy contributed to the coordinated expression of UCPs, we blocked autophagy in adipocytes using bafilomycin A1 and leupeptin, the established inhibitors of autophagosome acidification and lysosomal proteases, respectively.^25,38,39^ As expected, bafilomycin A1 and leupeptin potently attenuated autophagy as evidenced by p62 accumulation (Supplementary Figure 1). Intriguingly, inhibition of autophagy significantly increased UCP1 transcript (by 2.2-fold, *P*<0.001) but reduced the expression of UCP2 (by 38%, *P*<0.01) and UCP3 (by 89%, *P*<0.001) in adipocytes (Figures 3d–f), concomitant with suppression of adipogenesis (Figure 3g). These data recapitulated the effects of inhibiting FoxO1 on UCPs during adipocyte differentiation (Figure 2), thereby underlining the importance of FoxO1-autophagy axis in the coordinated expression of UCP1, UCP2 and UCP3.

### Nuclear localization and activity of FoxO1 was upregulated in differentiating adipocytes

**Figure 4:**
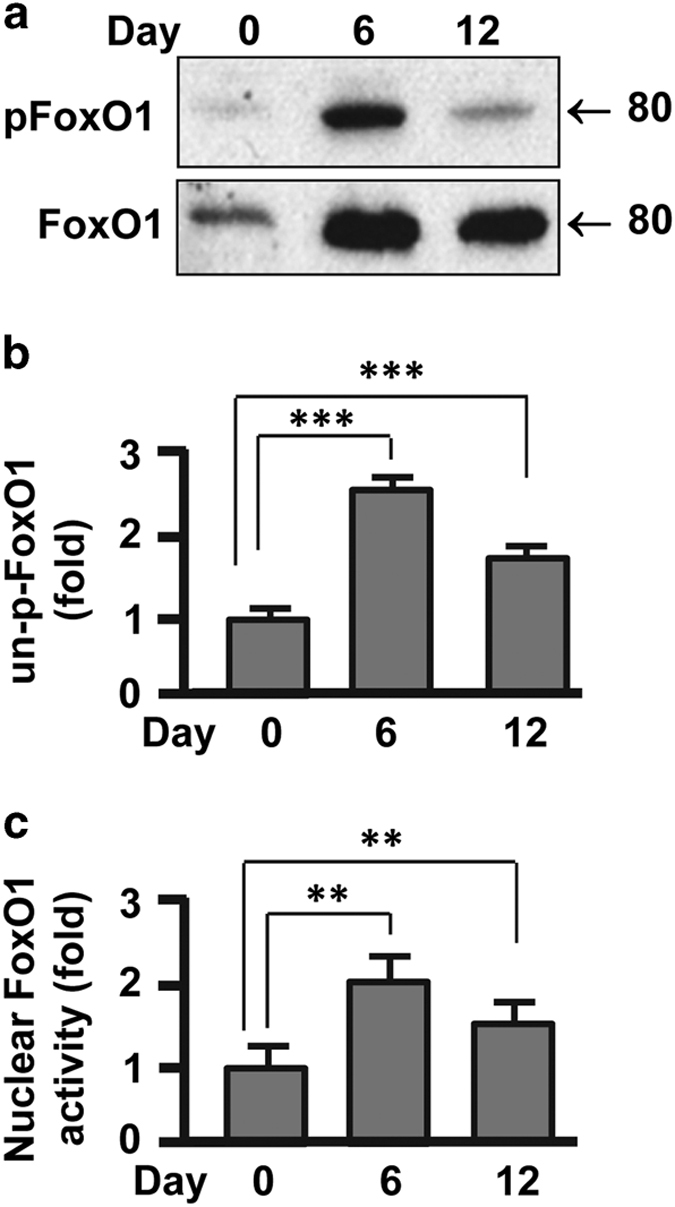
Nuclear localization and activity of FoxO1 increased during adipogenesis. (**a**) Western blot analysis of total FoxO1 and phosphorylated FoxO1 (pFoxO1-Thr24) during 3T3-L1 adipocyte differentiation. (**b**) Measurement of un-phosphorylated FoxO1 (un-p-FoxO1) by densitometric analysis of western blot images. (**c**) Measurements of FoxO1 activity in the nuclear fractions isolated from adipocytes on days 0, 6 and 12 during differentiation. Results were presented as mean±s.d.; *n*=3-4; **P<0.01; ***P<0.001.

Nuclear localization and activity of FoxO1 transcription factor is regulated by insulin-induced phosphorylation.^8,41^ To examine how insulin in the differentiation media affects FoxO1 distribution and activity, we measured total FoxO1 protein level and phosphorylated FoxO1 during adipocyte differentiation (Figures 4a and b). Intriguingly, FoxO1 underwent drastic upregulation during the cell differentiation, which significantly outweighed insulin-induced FoxO1 phosphorylation (Figure 4a). Indeed, densitometric analysis of western blot images confirmed that un-phosphorylated FoxO1 was increased during adipocyte differentiation, indicative of an increased distribution of nuclear FoxO1 (Figure 4b). To further validate this, we isolated nuclear fractions from preadipocytes (day 0), differentiating adipocytes (day 6) and differentiated adipocytes (day 12) for activity analysis. As shown in Figure 4c, FoxO1 activity was upregulated by 1.9-fold (*P*<0.01) and 1.5-fold (*P*<0.01) in the nuclear fractions from differentiating adipocytes and differentiated adipocytes, respectively, in comparison with that from preadipocytes. Therefore, nuclear distribution and FoxO1 activity was overtly increased during adipogenesis.

### FoxO1 regulated Tfeb by directly binding to its promoter

**Figure 5:**
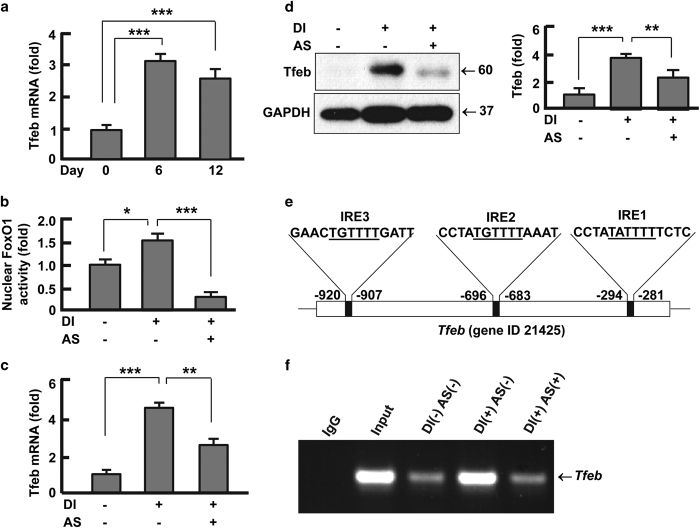
FoxO1 regulated Tfeb expression. (**a**) Tfeb transcript was analyzed on days 0, 6 and 12 during adipocyte differentiation. (**b**) FoxO1 inhibitor AS1842856 (0.1 μM) potently suppressed FoxO1 activity in the nuclear fractions isolated from adipocytes. (**c **and** d**) Inhibition of FoxO1 prevented Tfeb upregulation during adipocyte differentiation, both at transcript (**c**) and protein (**d**) levels. (**e**) Tfeb gene contains three FoxO1-binding (i.e., insulin response element, IRE) sites in its promoter region. (**f**) Chromatin immune-precipitation (ChIP) assay of FoxO1-Tfeb interaction using a FoxO1 specific antibody. DI, differentiation induction; AS, AS1842856. The cells were cultured and treated (days 0–12), and ChIP assay conducted as described in Materials and Methods section. Results were presented as mean±s.d.; *n*=3–4; *P<0.05; **P<0.01; ***P<0.001.

Tfeb has been shown to regulate both autophagosome and lysosome.^30^ Because Tfeb protein level and FoxO1 activity were coincidently upregulated during adipocyte differentiation (Figures 3 and 4), we asked the question whether Tfeb underwent transcriptional elevation during adipogenesis. By conducting qPCR analysis we found that Tfeb transcript was upregulated by 3.1-fold (*P*<0.001) and 2.5-fold (*P*<0.001) on day 6 and day 12, respectively (Figure 5a). Intriguingly, inhibition of FoxO1 led to significant suppression of Tfeb transcript (Figures 5b and c), accompanied with reduced abundance of Tfeb protein (Figure 5d). These results strongly suggest that FoxO1 is an upstream regulator of Tfeb. To examine whether FoxO1 interacts with Tfeb directly, we analyzed the promoter sequence in mouse Tfeb gene (gene ID 21425) and conducted chromatin immune-precipitation (ChIP) assay. As shown in Figure 5e, the promoter of Tfeb contains 3 insulin response elements, which function as specific binding sites for FoxO1 to interact with target genes.^21,41^ In addition, the abundance of Tfeb promoter bound to FoxO1 was higher in mature adipocytes than in preadipocytes (Figure 5f), in line with the increased distribution and activity of nuclear FoxO1 (Figure 4). Consistently, FoxO1 antagonist AS1842856 significantly reduced the abundance of Tfeb promoter that was bound to FoxO1 (Figures 5b and f). Therefore, FoxO1 directly regulates Tfeb gene expression through protein-DNA interaction.

## Discussion

FoxO1 and Tfeb have been implicated in autophagy regulation,^25,30,42,43^ but the interaction between these two transcription factors has not been reported. In this study we found that Tfeb was upregulated during adipocyte differentiation (Figure 3), concomitant with increased distribution and activity of nuclear FoxO1 (Figure 4). Importantly, FoxO1 directly bound to the promoter of Tfeb to achieve a transcriptional regulation (Figure 5). Inhibition of FoxO1 reduced both Tfeb transcript level and protein abundance, accompanied with downregulation of autophagy (Figures 2 and 5, and Supplementary Figure 1). Moreover, blockage of FoxO1-autophagy axis led to dysregulation of UCPs and suppression of adipocyte differentiation (Figures 1–3), suggesting that FoxO1-mediated autophagy is critical for coordinated expression of UCP1, UCP2 and UCP3 during adipogenesis. To our knowledge, this is the first report demonstrating the regulation of UCP2 and UCP3 by autophagy and its relation with FoxO1.

The differential UCP expression patterns during adipogenesis support the notion that UCP2 and UCP3 function differently from UCP1.^14,15,18,19^ UCP1 was gradually down-regulated during adipocyte differentiation (Figure 1), but inhibition of the FoxO1-autophagy axis upregulated UCP1 and significantly reduced lipid accumulation (Figures 2 and 3). It suggests that FoxO1-autophagy axis acts as a suppressor of UCP1, the physiological role of which may reside in preserving carbon source to support lipid synthesis for adipocyte maturation. Indeed, overexpressing UCP1 in adipocytes impairs oxidative phosphorylation but stimulates glycolysis and lactate production, which shunts carbon flux away from lipid synthesis and prevents lipid accumulation and adipocyte maturation.^34,35^ On the other hand, FoxO1-autophagy axis appeared to be critical for the induction of UCP2 and UCP3 as well as adipogenesis (Figures 2 and 3). Given that UCP2 and UCP3 regulated reactive oxygen species and lipid peroxide,^18,19,44^ the FoxO1-autophagy-UCP2/UCP3 axis may serve to maintain redox and lipid homeostasis that is critical for adipocyte differentiation.^23,45^ To this end, silencing of FoxO1 disturbs redox balance and prevents preadipocyte differentiation.^23^

The downstream pathway by which the FoxO1-autophagy axis differentially regulates UCPs remains to be defined. Although we cannot rule out the possibility that FoxO1 might directly regulate transcription of UCP genes, targeting FoxO1 or autophagy led to similar effects on UCP expression (Figures 2 and 3), corroborating an important role of the FoxO1→autophagy cascade in UCP regulation. Previous study suggested that suppression of autophagy by deleting Atg7 in skeletal muscle or liver promoted secretion of fibroblast growth factor 21 (FGF21), which in turn induced UCP1 in adipose tissue.^46^ It was also shown that suppression of autophagy reduced the stability of peroxisome proliferator-activated receptors γ (PPARγ),^40^ the key regulator of adipogenesis that also mediates UCP2 and UCP3 expression.^2,18^ To this end, we found that pharmacologically targeting the FoxO1-autophagy axis significantly reduced PPARγ level,^25^ which may account for the downregulation of UCP2 and UCP3 (Figures 2 and 3). Thus, future study examining the role of FGF21 and PPARγ in the regulatory network of FoxO1-autophagy axis will be of interest.

Taken together, our study demonstrates for the first time that FoxO1 induces the autophagy regulator Tfeb by binding to its promoter, and the FoxO1-autophagy axis differentially regulates UCP1, UCP2 and UCP3 in adipocytes. Given that obesity is linked to dysregulation of FoxO1,^2,41^ autophagy^38,47–49^ and UCPs,^5,10–12^ further studies of the FoxO1-autophagy-UCPs axis will advance our understanding of obesity and its related metabolic disorders.

## Materials and methods

### Materials

3T3-L1 preadipocytes (ATCC CL-173) were purchased from ATCC (Manassas, VA). Dulbecco’s modified Eagle’s (DMEM) medium was from Corning Inc (Manassas, VA). Fetal bovine serum (FBS) was from GeneMate (Kaysville, UT, USA). Dexamethasone, 3-isobutyl-1-methylxanthine (IBMX) and rosiglitazone were purchased from Cayman Chemical (Ann Arbor, MI, USA).+ Penicillin/streptomycin (P/S) was from GE Healthcare Life Sciences HyClone Laboratories (Logan, UT, USA). Insulin was from Sigma-Aldrich (St. Louis, MO, USA). FoxO1 inhibitor AS1842856 was from EMD Millipore (San Diego, CA, USA). Autophagy inhibitors bafilomycin A1 and leupeptin were from LC Laboratories (Woburn, MA, USA) and DOT Scientific Inc (Burton, MI, USA), respectively.

### Cell culture and treatment

3T3-L1 preadipocytes were cultured as previously described.^2,31^ In brief, the cells were cultured in basal media (DMEM media supplemented with 10% FBS, 100 units/ml penicillin and 100 μg/ml streptomycin (1×P/S)), at 37 °C in a humidified atmosphere of 5% CO_2_. The media were replaced every 2 days. 3T3-L1 preadipocytes were grown to confluence (day 0), and further maintained in fresh basal media for 2 days (days 1–2). At the end of day 2, the medium was changed to differentiation medium I: DMEM supplemented with 10% FBS, P/S (1×), IBMX (0.5 mM), dexamethasone (1 μM), insulin (1 μg/ml), and rosiglitazone (2 μM). At the end of day 4, the medium was changed to differentiation medium II: DMEM supplemented with 10% FBS, P/S (1 ×), and insulin (1 μg/ml). At the end of day 6, the medium was changed to basal media, and the cells were maintained in basal medium (replaced with fresh basal medium every 2 days) until they fully differentiated (day 12). Control preadipocytes were maintained in basal media and supplied with fresh medium every other day till day 12. Treatments with inhibitors (e.g., AS1842856 or bafilomycin A1 plus leupeptin) started on day 0 through day 12 (during differentiation) at the indicated concentrations.

### Measurement of lipid accumulation in adipocytes

Lipid accumulation in adipocytes was measured by oil red O staining.^2,25^ The oil red O working solution was freshly prepared by mixing 0.35% stock solution with dH_2_O (6:4) and filtered, and the staining was conducted on days 0, 6 and 12 as described.^2,25^ In brief, the media were removed and the cells were washed with phosphate-buffered saline (PBS), and fixed in 4% formaldehyde at room temperature for 10 min. Subsequently, the cells were washed with dH_2_O, and air dried completely. Oil red O working solution was added and the staining lasted for 1 h at room temperature. Afterwards, the stained cells were washed with dH_2_O for four times, and oil red O retained in the cells was extracted with isopropanol, and quantified by the absorbance at 510 nm on a Synergy H4 Hybrid Multi-Mode Microplate Reader (BioTek Instruments, Inc, Winooski, VT, USA).

### RNA extraction and cDNA synthesis

RNAs were extracted from cells with RNeasy Mini Kits (Qiagen, Germantown, MD, USA) according to the manufacturer’s instruction. The RNA samples were used to synthesize cDNA by reverse transcription PCR using iScript™ cDNA Synthesis Kits (Bio-Rad, Hercules, CA, USA) according to the manufacturer’s instruction.

### Real-time PCR

Gene expression was analyzed by quantitative real-time PCR on a ViiA 7 Real-Time PCR System (Life Technology, Grand Island, NY, USA).^1^ The primers used in this study were 5′- CAG CTT GCC TGG CAG ATA TCA-3′ (forward) and 5′- TTG GAT CTG AAG GCG GAC TT-3′ (reverse) for UCP1; 5′- TCT GCC CAG TCC CAT TCT CT-3′ (forward) and 5′- GGG AGG TGA GGT GGG AAG TAA-3′ (reverse) for UCP2; 5′- ACC TCC ATA GGC AGC AAA GGA-3′ (forward) and CGG AGG GCT GAA GTC CAA (reverse) for UCP3; 5′- CCA CCC CAG CCA TCA ACA C-3′ (forward) and 5′- CAG ACA GAT ACTCCC GAA CCT T-3′ (reverse) for Tfeb; and 5′- ACAGTCCATGCCATCACTGCC-3′ (forward) and 5′- GCCTGCTTCACCACCTTCTTG-3′ (reverse) for GAPDH as a reference gene.

### ChIP assay

ChIP assay was performed with an EZ-Magna ChIP A/G Chromatin Immunoprecipitation Kit (EMD Millipore, cat # 17–10086) as described previously.^21^ In brief, the cell culture was treated with 1% formaldehyde for 10 min, and the crosslinking reactions was stopped by adding glycine to a final concentration of 125 mM and incubating for 5 min at room temperature. Then the cells were rinsed with PBS, harvested in lysis buffer and incubate for 15 min. DNA was sheared and immunoprecipitation was conducted with a ChIP-grade anti-FoxO1 antibody (ab39670) from Abcam as described previously.^21^ Primers used to amplify the promoter of Tfeb were 5′- CCCCAAGTGGAAGTTGCTAA-3′ (forward) and 5′- ATGGCCCGTGATATGACTTT-3′ (reverse). PCR products were resolved by electrophoresis on 2.5% agarose gels.

### Measurement of nuclear FoxO1 activity

Nuclear fractions were isolated from cells using a TransAM Nuclear Extract Kit (Active Motif, cat # 40010), and FoxO1 activities were determined using a TransAM FKHR (FOXO1) Transcription Factor ELISA Kits (Active Motif, cat # 46396) according to the manufacturer’s instructions.

### Western blotting

To prepare cell lysates, the cells were washed with ice-cold PBS and homogenized using a Bullet Blender (Next Advance, Averill Park, NY, USA) in PLC lysis buffer (30 mM Hepes, pH 7.5, 150 mM NaCl, 10% glycerol, 1% Triton X-100, 1.5 mM MgCl_2_, 1 mM EGTA, 10 mM NaPPi, 100 mM NaF, 1 mM Na_3_VO_4_) supplemented with protease inhibitor cocktail (Roche), 1 mM PMSF.^2,31^ Total protein concentrations of the lysates were determined using the DC protein assay (Bio-Rad). Western blotting and image analysis were conducted as described previously.^2,31^ Antibody information: GAPDH (MA5-15738) and *β*-actin (MA5-15739) antibodies from Pierce (Rockford, IL, USA); antibodies against FoxO1 (9454 s), phospho-FoxO1 (Thr24) antibody (9464 s), LC3 (2775 s) and p62 (SQSTM1, 5114 S) from Cell Signaling Technology (Beverly, MA, USA); Tfeb (A303-673 A) antibody from Bethyl Laboratories, Inc. (Montgomery, TX, USA).

### Statistical analyses

All results were expressed as mean±s.d., and underwent analysis of variance to determine *P*-values; *P*<0.05 was considered statistically significant.

## Abbreviations

AS or AS1842856: 5-amino-7-(cyclohexylamino)-1-ethyl-6-fluoro-4-oxo-1,4-dihydroquinoline-3-carboxylic acid
Atg7: autophagy related 7
ATP: adenosine triphosphate
BL: bafilomycin-A1 and leupeptin
ChIP: chromatin immunoprecipitation
DI: differentiation induction
FBS: fetal bovine serum
FoxO1: forkhead box O1
FGF21: fibroblast growth factor 21
GAPDH: glyceraldehyde 3-phosphate dehydrogenase
LC3: microtubule-associated protein 1A/1B-light chain 3-phosphatidylethanolamine conjugate
p62: sequestosome 1 (SQSTM1)
PPARγ: peroxisome proliferator-activated receptor gamma
qPCR: quantitative polymerase chain reaction
Tfeb: transcription factor EB
UCP: uncoupling protein.
