# Supplementary figures and images for: FoxO1 interacts with transcription factor EB and differentially regulates mitochondrial uncoupling proteins via autophagy in adipocytes
Source: Cell Death Discov. 2016 Oct 3;2:16066–. doi: 10.1038/cddiscovery.2016.66 (PMC5046220; doi:10.1038/cddiscovery.2016.66)

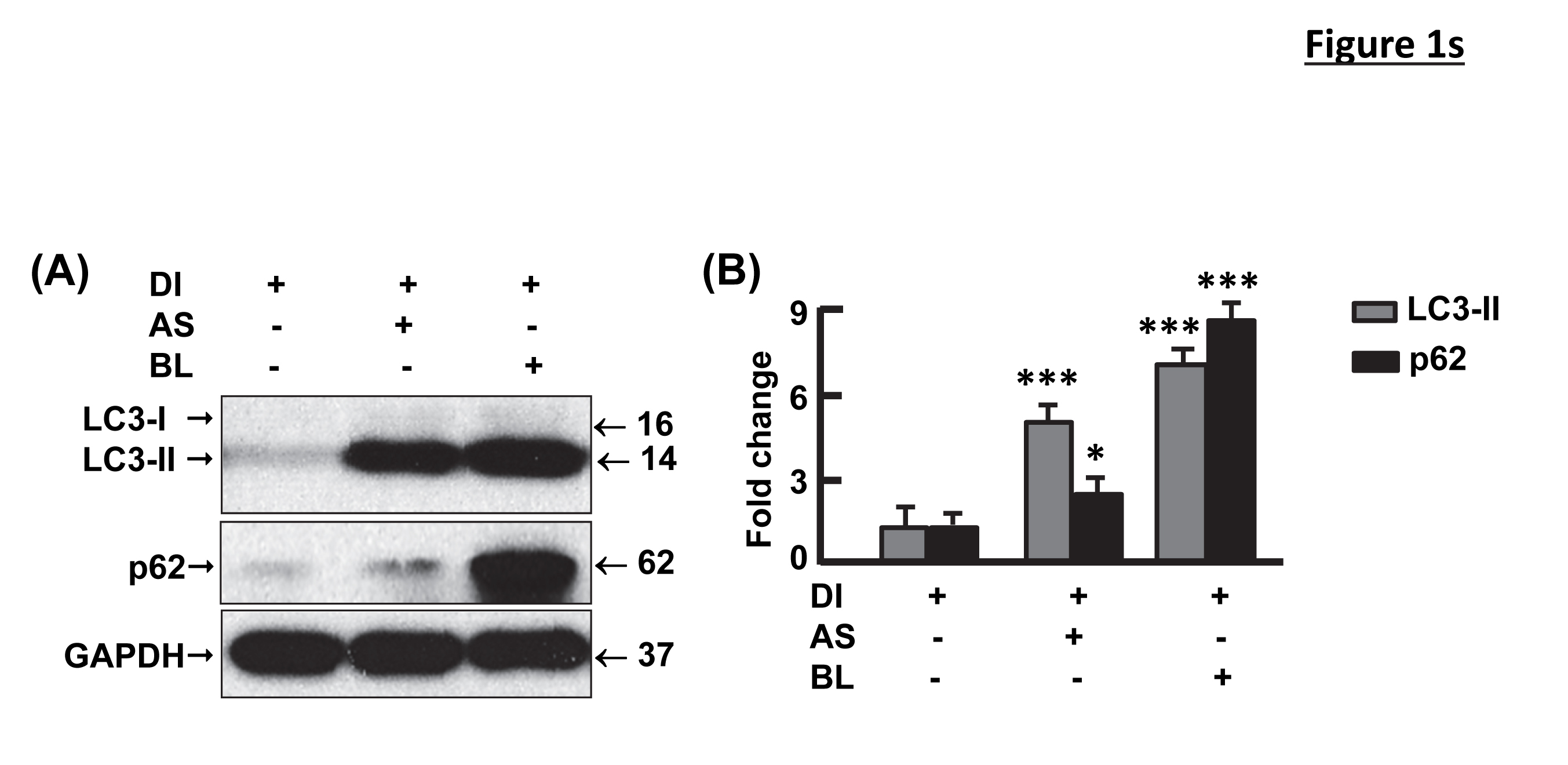

Supplement: Supplementary Information [file cddiscovery201666-s1.jpg]
